# Systematic Review: Is Intradiscal Injection of Bone Marrow Concentrate for Lumbar Disc Degeneration Effective?

**DOI:** 10.7759/cureus.9045

**Published:** 2020-07-07

**Authors:** Takashi Hirase, Robert A Jack, Kyle R Sochacki, Joshua D Harris, Bradley K Weiner

**Affiliations:** 1 Orthopedics and Sports Medicine, Houston Methodist Hospital, Houston, USA

**Keywords:** bone marrow concentrate, lumbar disc degeneration, degenerative disc disease, intradiscal injection

## Abstract

Current studies evaluating the outcomes of an intradiscal injection of bone marrow concentrate (BMC) for lumbar disc degeneration are limited. The purpose of this review was to determine if an intradiscal injection of BMC for lumbar disc degeneration results in a statistically significant improvement in clinical outcomes. A systematic review was performed using Preferred Reporting Items for Systematic Reviews and Meta-Analyses (PRISMA) guidelines. Levels I-IV investigations of intradiscal BMC injections in symptomatic lumbar disc degeneration were included in the analysis. Modified Coleman Methodology Scores (MCMS) were used to analyze study methodological quality. Only outcome measurements used by more than 50% of included studies, with a minimum follow-up of 12 months, were eligible for final data analysis. Pre-injection and post-injection visual analog scale (VAS) and Oswestry disability index (ODI) were compared using two-sample Z-tests. Seven articles (97 subjects (47 males, 38 females, 12 unspecified), mean age 33.9 ± 14.3 years, mean follow-up 44.4 ± 25.4 months) were analyzed. Six articles were level IV evidence and one article was level II. Mean MCMS was 56.6 ± 9.1. All subjects received single injections into the nucleus pulposus of one or more affected discs. VAS (66.0 mm to 20.9 mm; p<0.001) and ODI (44.4 to 19.1; p<0.001) significantly improved following the intradiscal BMC injection. One patient (1.0%) experienced herniated nucleus pulposus (HNP) following treatment. No other complications or re-injections were reported. In conclusion, despite our skepticism regarding the efficacy of the procedure, intradiscal injection of BMC for lumbar disc degeneration resulted in statistically significant improvement in VAS and ODI with low re-injection and complication rates in the studies assessed. Given that this study is limited to level IV evidence, the findings suggest that further randomized controlled studies may be worthwhile to evaluate the true efficacy of this treatment.

## Introduction and background

Lumbar disc degeneration is one of the most common causes of disability in the United States, felt to account for (directly or indirectly) over 40% of chronic low back pain [1-2]. Though intervertebral disc degeneration is usually an asymptomatic, normal, age-related phenomenon, it is recognized that it may be a source of acute or chronic low back pain [3-4]. A combination of various medical and physical treatments are often successful in treating such pain. However, over 10% of these patients are refractory to these non-surgical measures, occasionally requiring costly surgical procedures and contributing to public health concerns due to rising costs and health care use [5-6]. Current treatment options are limited despite the high prevalence and morbidity associated with disc degeneration. A combination of non-surgical measures, such as bed rest, non-steroidal anti-inflammatory drugs (NSAIDs), physical therapy, and analgesic injections, is a common treatment of early disease that has been shown to decrease symptoms but not slow down the potential progression of the disease [7-10]. Surgical procedures, such as spinal fusion, are often used for treating refractory disease but are invasive, expensive, and have high rates of postoperative complications [11-15].

In recent years, biological therapies have become increasingly popular, and the injection of bone marrow concentrate (BMC) into the intervertebral disc has emerged as a minimally invasive treatment option for patients unresponsive to non-invasive measures [16]. Mesenchymal stem cells comprise 0.01%-0.02% of BMC and have the ability to proliferate and differentiate into a variety of cell lineages, including cells within the nucleus pulposus and chondrocytes that make up the intervertebral discs [17-18]. MSCs have been shown to be successfully acquired in vivo from various sources, including bone marrow aspirate, adipose cells, and umbilical cord fragments [19-22]. Several animal studies have shown that the transplantation of 0.5 to 6 million MSCs within the nucleus pulposus via percutaneous or open procedures activates the further proliferation of nucleus pulposus cells while also stimulating the production of extracellular matrix proteins and collagen, which are vital to the supportive function of the intervertebral discs [19-22].

Though there are promising results among animal studies, current human studies evaluating the outcomes of intradiscal BMC injections in lumbar disc degeneration are mostly limited to small case reports and retrospective studies. Furthermore, there are currently no studies that investigate the clinical relevance of these outcomes. Thus, the purpose of this investigation was to determine if the intradiscal injection of BMC for lumbar disc degeneration results in a statistically significant improvement in clinical outcomes.

## Review

Methods

**Figure 1:**
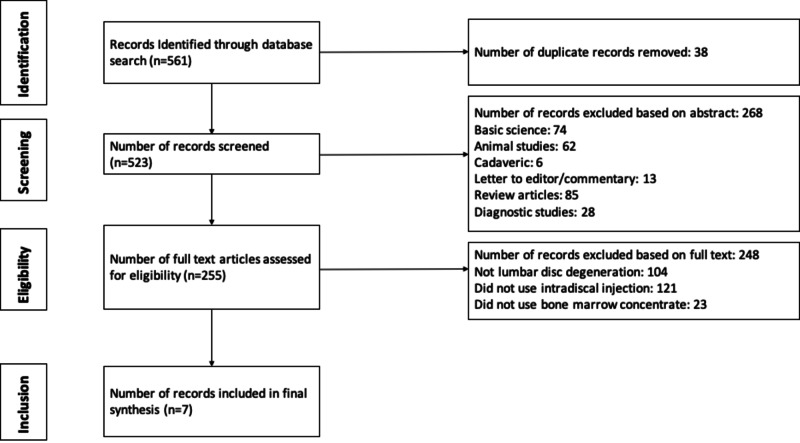
Flow diagram summarizing the literature search, screening, and review

A systematic review was registered with the International Prospective Register of Systematic Reviews (PROSPERO) on October 26, 2017 (Registration # CRD42017075842). Preferred Reporting Items for Systematic Reviews and Meta-analyses (PRISMA) guidelines were followed and a checklist created [23]. Eligible studies consisted of levels I-IV (via Oxford Centre for Evidence-Based Medicine (CEBM)) therapeutic studies that investigated the outcomes of intradiscal BMC injections for lumbar disc degeneration among adult human patients [24]. The diagnosis was made in each included study based on a combination of history, physical examination, and radiographs, including magnetic resonance imaging (MRI). Pfirrmann grade, modified Pfirrmann score, decreased fluid density, the presence of intervertebral regressive degeneration, and/or the presence of a posterior disc bulge were used to classify the severity of degeneration included in each study. Studies that included other etiologies of back pain were excluded. Cadaveric studies, basic science and animal studies, diagnostic studies, economic studies, prognostic studies, level V evidence expert opinion, letters to editors, and review articles were also excluded. Studies published in non-English languages were eligible for inclusion (none were identified in search conduct). In cases of different studies with duplicate subject populations, the study with the longer follow-up, higher level of evidence, greater number of subjects, or greater clarity of methods and results was included. The authors conducted separate searches of the following medical databases: MEDLINE, Web of Science, and Cochrane Central Register of Controlled Trials databases. Under the PROSPERO registration, similar prior systematic reviews and meta-analyses were sought and none were identified. The searches were performed on April 24, 2020. The search terms used were “mesenchymal stem cells”, “bone marrow concentrate”, “degenerative disc”, “spine”, and “injection.” The search results were reviewed for duplicates and the inclusion criteria determined the articles that were included in the final analysis (Figure 1).

Two authors independently reviewed all articles using the methodology recommended by Harris et al. [25]. The study design, patient populations, and procedure technique were first identified. All lower back-specific patient-reported outcome scores, re-injection rates, and complication rates were analyzed.

The levels of evidence were then assigned based on the Oxford Centre for Evidence-Based Medicine [26]. Study methodological quality was analyzed using the Modified Coleman Methodology Score (MCMS) and was considered good with scores between 70 and 84, fair with scores between 55 and 69, and poor with scores less than 55 [27]. Based on the quality of the evidence, the overall strength-of-recommendation taxonomy (SORT) and grading of recommendations assessment, development and evaluation (GRADE) scores were provided [28-29]. Study heterogeneity and nature of evidence (mostly retrospective, non-comparative) precluded meta-analysis. Thus, a best-evidence synthesis was used instead [30]. Only the outcome measurements used by 50% or more of the studies were included in the data synthesis to increase the power of the measurement over that of individual studies. A weighted mean of pre-injection and post-injection values from each study were calculated and comparisons were made using two-sample Z-tests using a p-value of less than 0.05 for significance.

Results

Seven articles were analyzed (Table 1) [31-37]. Six articles were level IV evidence and one article was level II. Three studies were performed in the United States and two studies each were performed in Japan and Spain. According to MCMS, one article was good, four articles were fair, and two articles were poor. The mean MCMS was 56.6 ± 9.1. The overall SORT score was B and the GRADE score was C. There was a total of 97 patients analyzed. There were 47 males, 38 females, and 12 unspecified genders. The mean age was 33.9 ± 14.3 years old, with a mean follow-up of 44.4 ± 25.4 months. BMC was obtained by centrifugation of 30 to 90 mL of autologous (six studies) bone marrow aspiration from the iliac crest or posterior superior iliac spine (PSIS) to perform a fluoroscopy-guided injection of 2 to 3 mL of BMC directly into one or more symptomatic intervertebral discs. The estimated number of MSCs injected per disc ranged from 1x10^6^ to 3.6 x 10^8^ cells.

**Table 1:** Study demographics NR (not recorded); M (male); F (female); BMC (bone marrow concentrate); FRI (functional rating index); SF-36 (36-item short-form health survey); SF-12 (12-item short-form health survey); SANE (single assessment numeric evaluation); VAS (visual analog score); ODI (Oswetry disability index); QOL (quality of life); JOA (Japanese Orthopaedic Association scoring system); QOL (quality of life); PSIS (posterior superior iliac spine)

Study	Orozco et al. 2011	Yoshikawa et al. 2010	Pettine et al. 2017	Mochida et al. 2015	Noriega et al. 2017	Centeno et al. 2017	Elabd et al. 2016
Type of study	Case Series	Case Series	Case Series	Case Series	Prospective randomized control study	Case Series	Case Series
Level of evidence	IIA	IV	IV	IV	II	IV	IV
Country	Spain	Japan	USA	Japan	Spain	USA	USA
No. subjects	10	2	26	9	12	33	5
Gender (M/F)	4/6	0/2	11/15	8/1	NR	21/12	3/2
Age (Mean)	35	68.5	40	25.7	NR	40.3	40.4
Classifications of degenerative discs included	Discs with decreased fluid density with preserved external annulus fibrosus (Adams stages 2-4)	Discs with intervertebral regressive degeneration and instability	Modified Pfirrmann scores 4-7	Pfirrmann grade III	Pfirrmann grades II-IV	Degenerative discs with posterior disc budge	Degenerative discs with posterior disc bulge ≥ 2 mm
Source of BMC	Autologous iliac crest	Autologous iliac crest	Autologous iliac crest	Autologous iliac crest	Allogeneic iliac crest	Autologous left PSIS	Autologous left PSIS
Amount injected	5x10^6^ cells per disc from a suspension containing 10^7^cells/ml	10 ml of 10^5^cells/ml in combination with collagen sponge	2-3 ml per disc; 1.2x10^8^ cells per ml	1x10^6^ cells/702 𝜇L sterile saline per disc	25x10^6^ cells per disc from a suspension containing 12.5 x 10^6 ^cells/ml	1-3 ml per disc; avg 2.3x10^7^ cells per disc	0.25-1 ml per disc; avg 30.8 x 10^6^ cells per disc
Injection method	Fluoroscopy guided intradiscal injection	Fluoroscopy guided intradiscal injection	Fluroscopy guided intradiscal injection	Fluoroscopy guided intradiscal injection	Fluoroscopy guided intradiscal injection	Fluoroscopy guided intradiscal injection	Fluoroscopy guided intradiscal injection
Follow-up (months)	12	24	36	36	12	72	72
Outcomes	ODI, VAS, SF-36	VAS, JOA	ODI, VAS	JOA	ODI, VAS, SF-12	VAS, FRI, SANE	QOL Questionnaire

All studies performed intradiscal BMC injections after the failure of non-interventional management. One study used a negative control with a mepivacaine injection, which reported a significant decrease in visual analog score (VAS) and Oswestry disability index (ODI) eight days after the injection as compared to pre-injection. However, the study did not report any significant changes in VAS nor ODI at three, six, or 12 months post-injection as compared to pre-injection. No comparison injections were made in any of the other studies. There was one case (1.0%) of herniated nucleus pulposus (HNP) months post-injection. Though no re-injections were performed, six patients (6.2%) eventually required surgical management due to unresolved pain. No other complications or re-injections were reported (Table 2).

**Table 2:** Individual study outcome measures NR (not recorded); F/U (follow-up); SF-36 (36-item short-form health survey); SF-12 (12-item short-form health survey); VAS (visual analog score); ODI (Oswetry disability index); JOA (Japanese Orthopaedic Association scoring system); FRI (functional rating index); SANE (single assessment numeric evaluation); QOL (quality of life); HNP (herniated nucleus pulposus)

Study		Orozco et al. 2011	Yoshikawa et al. 2010	Pettine et al. 2017	Mochida et al. 2015	Noriega et al. 2017	Centeno et al. 2017	Elabd et al. 2016
SF-36	Baseline	12.7 ± 3.7	NR	NR	NR	NR	NR	NR
	Final F/U	24.8 ± 3.9	NR	NR	NR	NR	NR	NR
SF-12	Baseline	NR	NR	NR	NR	46 ± 3	NR	NR
	Final F/U	NR	NR	NR	NR	48 ± 3	NR	NR
VAS	Baseline	68.9 ± 3.3	NR	82.1 ± 2.6	NR	67 ± 7	52	NR
	Final F/U	20.0 ± 6.5	28.0	21.9 ± 4.4	NR	47 ± 10	20	NR
ODI	Baseline	25.0 ± 4.1	NR	56.7 ± 3.6	NR	34 ± 7	NR	NR
	Final F/U	7.4 ± 2.3	NR	17.5 ± 3.2	NR	22 ± 7	NR	NR
JOA	Baseline	NR	2.5	NR	14.2 ± 4.8	NR	NR	NR
	Final F/U	NR	17.0	NR	27.2 ± 1.6	NR	NR	NR
FRI	Baseline	NR	NR	NR	NR	NR	61	NR
	Final F/U	NR	NR	NR	NR	NR	12	NR
SANE	Baseline	NR	NR	NR	NR	NR	42	NR
	Final F/U	NR	NR	NR	NR	NR	60	NR
QOL	Baseline	NR	NR	NR	NR	NR	NR	N/A
	Final F/U	NR	NR	NR	NR	NR	NR	55% Improvement
Complications		0	0	0	0	0	1 - HNP	0
Re-Injection		0	0	0	0	0	0	0
Eventual surgical management		0	0	6	0	0	0	0

Mean VAS decreased by 41.2 mm, 45.7 mm, 45.1 mm, and 48.8 mm at three, six, 12, and 24 months following the intradiscal BMC injection, respectively. Mean ODI decreased by 26.9, 27.1, 25.3, and 26.1 at three, six, 12, and 24 months following the intradiscal BMC injection, respectively (Table 3; all p <0.001 vs baseline).

**Table 3:** Average study outcome measures included in the best-evidence synthesis NR (not recorded); F/U (follow-up); VAS (visual analog score); ODI (Oswestry disability index); *P<0.001 vs baseline

	VAS (mm)	ODI
Baseline	66.0 ± 12.3	44.4 ± 16.3
3-month F/U	24.8 ± 11.1*	17.5 ± 3.5*
6-month F/U	20.3 ± 11.4*	17.3 ± 5.9*
12-month F/U	20.9 ± 17.2*	19.1 ± 8.5*
24-month F/U	17.2 ± 8.2*	NR

Discussion

This systematic review suggests that intradiscal injection of BMC for lumbar disc degeneration resulted in a statistically significant improvement in VAS and ODI with low re-injection and complication rates for the reported studies. The methodological quality of the studies was fair. To our knowledge, this is the first systematic review to evaluate the outcomes of intradiscal injection of BMC for disc degeneration. The findings were contrary to our expectations.

All studies analyzed utilized intradiscal injection of BMC to treat both the symptoms and the progression of disc degeneration. A study by Pang et al., which was excluded from this review, utilized allogeneic umbilical cord tissue to harvest the MSCs [38]. The authors hypothesized that human umbilical cord tissue-derived mesenchymal stem cells (HUC-MSCs) contain stem cells that can regenerate degenerative discs utilizing the same mechanisms but with additional capacity for localized immunosuppression. This study of two patients resulted in an average VAS and ODI decrease of 50.0 mm and 38.5, respectively, at the 24-month follow up with no adverse effects.

Surgical rates following intradiscal BMC injection were low (6.2%) and might be compared to reoperation rates of 9.1% following spinal fusion or 7.8% for disc arthroplasty as reported by Jacobs et al. [39]. All six cases requiring eventual surgical management were reported in a single study by Pettine et al., and no other study reported required surgical management [34]. Reported complication rates after BMC injection were low. Besides one case of post-injection disc herniation following treatment, there were no reported adverse effects reported. Again, this complication rate after BMC injection may be compared to 5.0% in spinal fusions and up to 16.7% in disc arthroplasty for similar populations [40].

There are several important limitations among the studies included in this review. Six of the seven articles were level IV evidence, which limits the strength of the results [31-36]. None of the studies used a double-blinded approach, producing potential bias. The average study methodological quality as assessed by the MCMS was fair. The assimilation of heterogeneous low methodological quality studies with VAS and ODI is a significant limitation. We aimed to minimize this as much as possible with strict study eligibility and inclusion criteria, despite the level IV evidence nature of the studies. The heterogeneity of outcome measures used among the studies limited the data analysis to two outcome measures. Additionally, significant heterogeneity in BMC sources (age difference, specifically) may have affected the quality of MSCs. Donor site morbidity may be another limitation that may have led to a lower significant decrease in post-injection pain VAS and ODI. Variable restrictions and regulations among different nations in manipulating MSCs might have also impacted outcomes. Prospective comparative trials, with greater study sizes, using standardized, validated clinical outcome measures are needed.

## Conclusions

Despite our skepticism regarding the efficacy of the procedure, intradiscal injection of BMC for lumbar disc degeneration resulted in a statistically significant improvement in VAS and ODI with low re-injection and complication rates in the studies assessed. Given this study is limited to level IV evidence, the findings suggest that further randomized controlled studies may be worthwhile to evaluate the true efficacy of this treatment.
